# Digging out the Molecular Connections between the Catalytic Mechanism of Human Lysosomal α-Mannosidase and Its Pathophysiology

**DOI:** 10.1021/acs.jcim.4c02229

**Published:** 2025-02-20

**Authors:** Bruno Di Geronimo, Špela Mandl, Santiago Alonso-Gil, Bojan Žagrović, Gilbert Reibnegger, Christoph Nusshold, Pedro A. Sánchez-Murcia

**Affiliations:** † Laboratory of Computer-Aided Molecular Design, Division of Medicinal Chemistry, Otto-Loewi Research Center, Medical University of Graz, Neue Stiftingtalstr. 6/III, A-8010 Graz, Austria; ¶ Max Perutz Labs, Vienna Biocenter Campus (VBC), Campus Vienna Biocenter 5, 1030 Vienna, Austria; § Department of Structural and Computational Biology, Vienna Biocenter, University of Vienna, Campus Vienna Biocenter 5, A-1030 Vienna, Austria; ∥ BioTechMed-Graz, Mozartgasse 12/II, A-8010 Graz, Austria

## Abstract

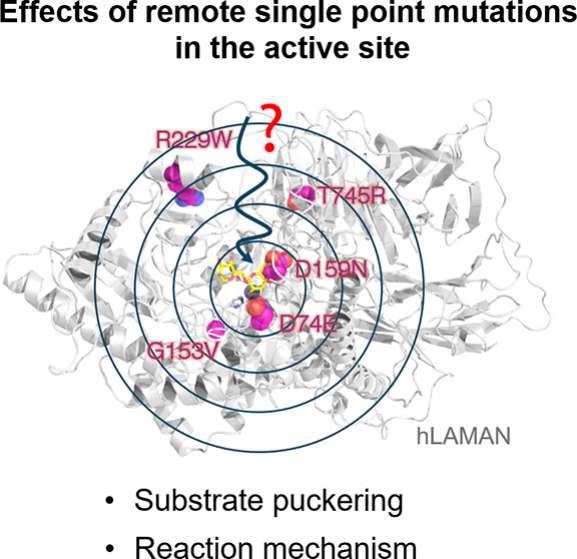

Human lysosomal α-mannosidase (hLAMAN) is a paradigmatic example of how a few missense mutations can critically affect normal catabolism in the lysosome and cause the severe condition named α-mannosidosis. Here, using extensive quantum mechanical/molecular mechanical metadynamics calculations, we show how four reported pathological orthosteric and allosteric single-point mutations alter substrate puckering in the Michaelis complex and how the D74E mutation doubles the energy barrier of the rate-limiting step compared to the wild-type enzyme.

## Introduction

Human lysosomal α-mannosidase (hLAMAN, EC 3.2.1.24) belongs to the Zn^2+^-dependent aspartic glycosidase hydrolase family GH38.^1−4^ hLAMAN has a large globular domain (962 amino acids) with an active site centered on the helix core. The enzyme catalyzes the cleavage of end-terminal mannosidic linkages α(1 → 2), α(1 → 3), and α(1 → 6) from oligosaccharides. The total or partial loss of hLAMAN activity caused by missense mutations is accompanied by the diagnosis of the rare genetic disorder α-mannosidosis (MANSA).^5−7^ MANSA belongs to the lysosomal storage disorders (LSDs), and although it has a very low incidence (1 in 500,000 live births), the current estimation is considered to be higher due to a large number of undiagnosed patients.^7,8^ MANSA is characterized by symptoms such as intellectual disability, unusual facial features, or skeletal anomalies, among others.^9,10^ Despite the remarkable severity of some of the MANSA symptoms, no permanent cure is available yet. Bone marrow transplantation and enzyme replacement therapy, via administration of the recombinant enzyme (i.e., velmanase alfa, Lamzede), are the two established available therapies for handling MANSA, which are associated with multiple side effects and risks.^11,12^

**Figure 1:**
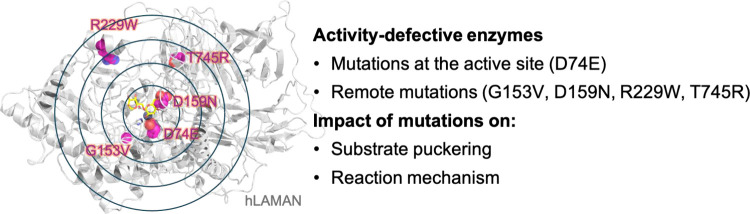
Investigation of the impact of proximal and remote mutations on the hLAMAN reaction mechanism.

Disease-associated variants can exhibit alterations in protein flexibility, substrate/cofactor and inhibitor binding, or their post-translational modification pattern.^13−16^ In MANSA, more than 130 pathogenic enzyme variants have been detected and classified:^5−7,17−20^ 23 are caused by point mutations described to alter the normal intracellular processing, trafficking to the lysosome, and/or the 3D structure of the enzyme. Interestingly, 17 missense mutations are known to cause a significant loss of enzymatic activity (Table S1). We previously studied their impact on the stability of hLAMAN, their connectivity with the active site of the enzyme, and their role in dynamics using conformational ensembles.^21^ In our analysis, we found that mutations affecting the enzyme activity (i.e., G153V, D159N, R229W, and T745R) tend to be significantly coupled with the residues of the active site (Figure S1). Thus, these reported mutations seem to affect the amino acid network from their remote position to the active site. But, how? Herein we want to answer this question. We study the impact of these remote mutations on the geometry of the Michaelis complex as well as the impact of the orthosteric mutation D74E on the energy barrier of the first step of the catalytic reaction by hLAMAN using extensive quantum mechanics/molecular mechanics (QM/MM) metadynamics calculations. Since most of the analyzed mutations are located beyond 10 Å away from the active site, this work is aimed to show the versatility of QM/MM metadynamics to see in all-atom detail changes at the active site of the enzyme upon mutation (Figure 1).

## Methods

### System Preparation

The structure of the human lysosomal α-mannosidase (hLAMAN, UniProtKB - O00754) was taken from the AlphaFold Protein Structure Database (AF-O00754-F1).^23^ The confidence obtained for the deep-learning model is extremely high for almost all residues in the structure of hLAMAN except for the N-terminal lysosomal signal peptide (residues 1–49), which was not included in our simulations. The oligosaccharides for the N-glycosylation points at positions N137, N497, N645, N692, and N766, as well as the catalytic zinc atom, were taken from the crystal structure of the bovine lysosomal α-mannosidase (bLAMAN) after structural superimposition (1O7D, UniProtKB - Q29451).^2^ Additionally, two N-acetyl glucosamine moieties were added to positions N367 and N930. The experimentally reported disulfide bonds for the pairs 55-358, 268-273, 412-472, and 493-5019, were already present in the AlphaFold2 model and were included when building up the topology of the system. The protonation states of the titratable residues on the protein were calculated via the H++ web server assuming a pH value of 4.8.^24^ Care was taken with the catalytic residue D319 (general acid for the first step of the reaction), which was manually protonated to accomplish the proposed mechanism of action. The structure of the substrate α-d-mannopyranosyl-(1→6)-β-d-mannopyranose (α-mannobiose, AMB) was taken from the crystal structure of one GH125 1,6-α-mannosidase variant in complex with the former substrate (PDB id. 5M7I)^25^ and manually docked into the active site of hLAMAN after structural superimposition, guided by the reference compound TRS in the crystal of bLAMAN (PDB id. 1O7D).^25^ It must be stressed that the crystallographic mannose ring −1 of AMB was found to have a ^O^*S*_2_ conformation. The rest of the system was treated with the force field ff19SB^26^ and GLYCAM06^27^ for the glycosidic residues and substrate.^27^ The 5 simulated hLAMAN variants discussed in this work (D74E, D159N, G153V, R229W, and T745R) were generated with AlphaFold2^23^ as well.

All systems were first prepared for the QM/MM MD simulations following a general protocol of classical MD simulations as described before.^21^ Briefly, all systems were immersed in a TIP3P water box with 28,000–29,000 TIP3P water molecules,^28^ depending on the system. Each ES complex was minimized in three steps, where hydrogen atoms, solvent molecules and counterions, and the solute were sequentially allowed to relax. Once minimized, each system was heated from 100 to 300 K in an NVT ensemble using the Langevin thermostat (friction coefficient gamma of 1.0 ps^–1^). Care was taken to constrain the solute during the heating step by imposing a harmonic force on each atom of the solute of 40 kcal mol^–1^ Å^–2^. Subsequently, these harmonic constraints were gradually reduced to a value of 10 kcal mol^–1^ Å^–2^ in four simulation stages (NVT, 300 K). Then, the systems were switched to constant pressure (NPT scheme, Berendsen barostat^29^ at 300 K), and the imposed constraints from the heating were completely removed. For the MM part, the SHAKE algorithm^30,31^ was applied to restrain the hydrogen atoms, and the time step was set at 1 fs. The final geometries of the solvated and equilibrated systems were further simulated for 0.5 μs. Some structural comparisons between these initial geometries for metadynamics can be found in Table S5. All calculations were run using Amber20^32^ on NVIDIA GTX3090Ti GPUs.

### Well-Tempered QM/MM Metadynamics

The computed energy surfaces in this work correspond to simulations using one initial MD snapshot. As selection criteria of this snapshot, we paid attention to key distances between atoms for catalysis for the nucleophilic attack and proton transfer. All QM/MM MD simulations were performed using Amber20^32^ coupled to TeraChem^33−36^ and Plumed 2.7.^37,38^ The QM region includes the coordination sphere of Zn^2+^ (D196, D74, H72, and H445 side chains starting from β-carbons), the AMB substrate (corresponding disaccharide of the reaction), and the side chain from D319 (acid/base catalyst); 87 atoms in total (Figure S2). The QM region was treated with the generalized gradient approximation (GGA) functional PBE^39,40^ with a double-ζ (DZ) def2-SVP quality basis set^41^ except the Zn^2+^ metal ion, which was treated with LANL2DZ pseudopotentials.^42^ We employed electrostatic embedding in the QM/MM calculations. We computed the long-range QM-QM and QM-MM electrostatic interactions with particle mesh Ewald (PME)^43^ to ensure accurate and efficient handling of long-range electrostatic interactions. In the QM/MM simulation, the classical valence terms crossing the QM/MM boundary were treated using the default link atom scheme in the QM/MM Amber interface. Preliminary simulations conducted within semiempirical methods within the QM region were ran with the self-consistent-charge density-functional tight-binding method (DFTB3).^44^ The rest of the system (MM partition) was modeled using the ff19SB force field^26^ for the overall protein structure and the GLYCAM06^27^ for the associated N-glycosylated post-translational modifications (PTMs). Parameters for all atoms inside the coordination sphere of the catalytic zinc were parametrized using the Metal Center Parameter Builder (MCPB) approach.^45^ The geometry optimization of the Zn^2+^-coordination sphere in the gas phase was performed using Gaussian16 v. C.01^46^ at the B3LYP/6-31G* level of theory.^47−50^ Before the production phase of all QM/MM metadynamics, an initial 5 ps simulation step without collective variable definition was run and not included in the analysis.

**Figure 2:**
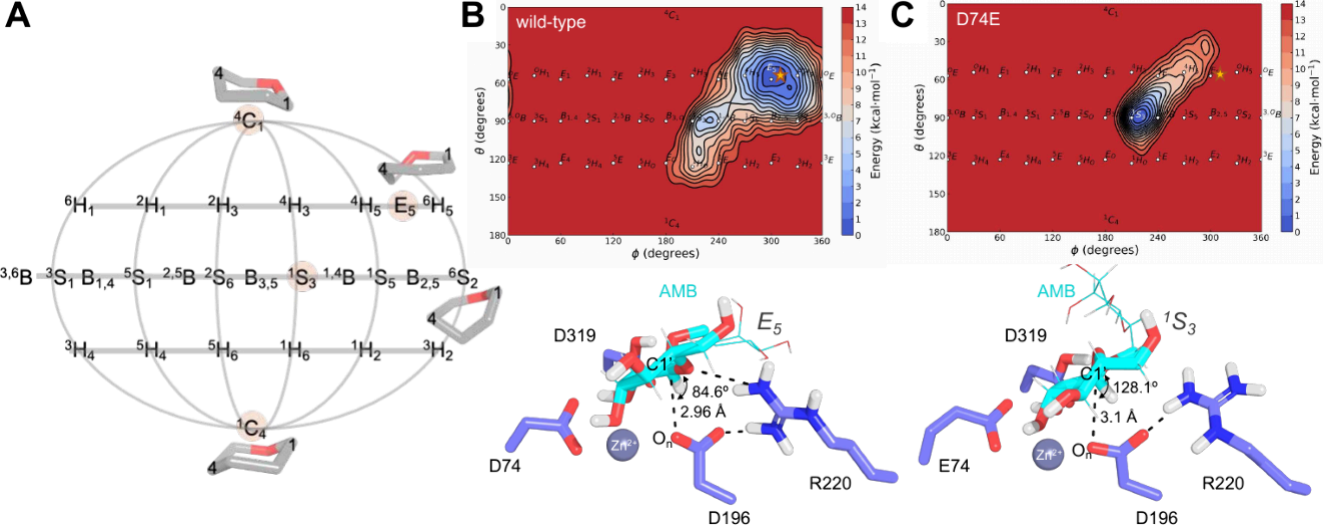
(A) Schematic representation of the 2D projection of the Cremer–Pople sphere. Conformations ^4^*C*_1_, *E*_5_, ^1^*S*_5_, and ^1^*C*_4_ are highlighted. (B) Conformational 2D free energy surface (kcal mol^–1^) for the puckering of the mannose ring −1 in AMB at the active site of wild-type hLAMAN. Detail of the wild-type ES complex at the energy minimum. (C) Conformational 2D free energy surface (kcal mol^–1^) for the puckering of the −1 ring of the AMB at the active site of D74E hLAMAN. Detail of the D74E ES complex at the energy minimum. The star represents the α-mannose conformation from the 3BUP crystallographic structure.^22^ The mannose ring +1 of AMB is shown as lines for the sake of clarity.

**Figure 3:**
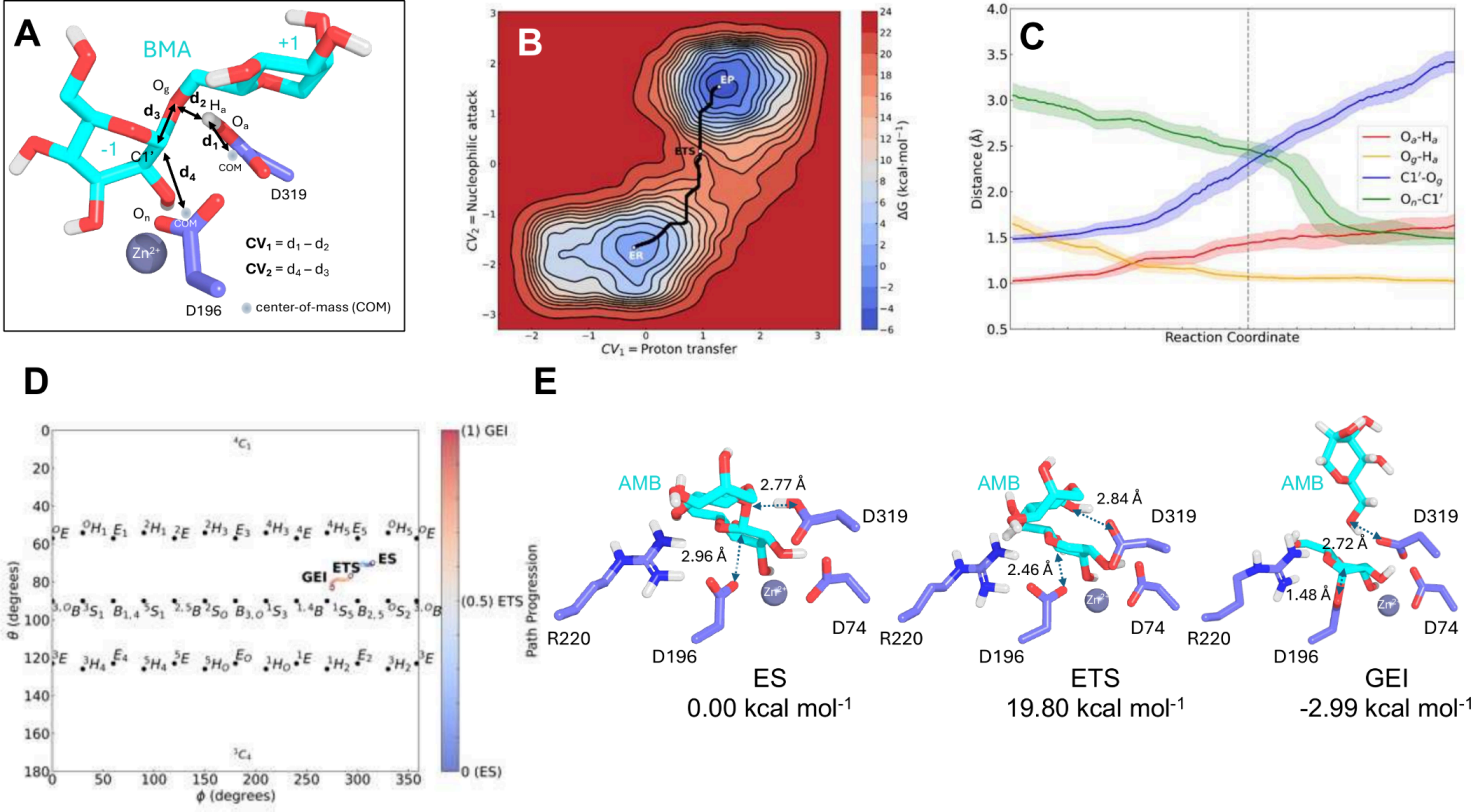
(A) Definition of collective variables CV_1_ and CV_2_. (B) 2D free energy surface for the catalyzed reaction by wild-type hLAMAN. Black dots represent MFEP. ES complex, ETS complex, and GEI are highlighted. (C) Evolution of catalytic distances (Å, running averages over 0.2 Å data points) along the MFEP: O_*a*_–H_*a*_ (red), H_*a*_–O_*g*_ (orange), C1′–O_*g*_ (blue), and O_*n*_–C1′ (green). The ETS is highlighted with a dotted line. (D) Puckering evolution along the reaction. (E) Representative average structure along the MFEP for the ES complex, ETS complex, and GEI of wild-type hLAMAN.

- Puckering analysis. In the QM/MM metadynamics,^51^ for the puckering analysis of the d-mannose residue at position −1 in the substrate AMB of each variant, the Cremer–Pople parameters ϕ and θ angles were used as collective variables. All simulations started from an equilibrated snapshot from the previous MD simulations. A well-tempered metadynamics^52^ with a bias factor at 15 kcal mol^–1^ was run for all systems. The height and width of the Gaussian terms were set at 0.75 kcal mol^–1^ and 0.1 rad, respectively. All simulations were stopped after they reached 14 kcal mol^–1^. The total hills added for each system were wild-type (802), D74E (321), G153V (847), D159N (410), R229W (466), and T745R (540). In the studies conducted using the DFTB3 semiempirical method, the wild-type was simulated for 57 ps, during which a total of 763 hills were added to the metadynamics simulation. The mutant D74E was simulated for 99 ps, with 2001 hills incorporated into the simulation. The results for the different enzyme variants are shown in Figure 2 and Figure S8.
- Reaction mechanism. In the context of reaction mechanism studies, two collective variables (CV_1_ and CV_2_, see Figure 3A) were chosen to describe the proton transfer from D319 and the glycosidic oxygen (O_*g*_, CV_1_) and the glycosidic bond cleavage and the nucleophilic attack by D196 (CV_2_). CV_1_ is composed of the difference between *d*_1_ (distance between the center-of-mass (COM) of the carboxylic oxygen atoms in D319 and the proton H_*a*_ on the same residue) and *d*_2_ (distance between the former H_*a*_ atom and the glycosidic oxygen O_*g*_ in AMB). CV_2_ is defined as the difference between *d*_3_ (distance between the O_*g*_ oxygen of the ring +1 and the former C1′ of the −1 ring) and *d*_4_ (distance between the anomeric carbon C1′ and the COM of the carboxylate oxygen atoms in D196). For the wild-type, the height and width of the Gaussian terms were set to 1.0 kcal mol^–1^ and 0.15 Å for both collective variables, and Gaussian-like potentials were added every 75 time steps with a bias factor set to 25 kcal mol^–1^. For the D74E mutant, the same method was applied with the exceptions that the bias factor was set to 75 kcal mol^–1^ and the height was set to 2.5 kcal mol^–1^. We defined energy walls for each of the two CVs at appropriate distances to avoid unsuccessful exploration events far away from the chemical event. The total number of Gaussian terms added was 1776 for the wild-type system and 1232 for D74E. The reaction coordinate was taken from the minimum free energy pathway (MFEP), computed with the MEPSA program.^53^ FEPs were considered converged once the system evolved from reactants to products and returned to reactants again (one cycle). The results for the energy profile of the enzymatic reaction are shown in Figure 3 and Figure S6.

Geometries of the ES complex, ETS complex, and GEI (whole system without water molecules and counterions) as PDB files for both wild-type and D74E variants can be found at 10.5281/zenodo.14251812.

### Reactive and Nonreactive Conformations via Δ*G*_*r*/*nr*_

To quantify the number of reactive and nonreactive conformations during the QM/MM metadynamics, we use here the term Δ*G*_*r*/*nr*_, which refers to the ratio to free energies of reactive and nonreactive explored conformations. To identify reactive and nonreactive conformations, we have made use of the previous calculation of the puckering coordinates (ψ, θ) of several transition state mimic inhibitors inside the active site of some families of glycoside hydrolases (see Figure 7 of ref (54)). Conformations on the north part of the plot between the conformation ^4^*C*_1_ and the line defined by the explored transition state mimetics are considered reactive conformations. Those between this line and the ^1^*C*_4_ conformation (southern part) are considered nonreactive.

### Solvent-Accessible Surface Area (SASA) Calculations

The SASA ratio of the substrate (SASAsub) to the active-site pocket (SASApkt) and the substrate-positioning index (SPI)^55^ were calculated using the QM/MM-puckering trajectories. SASAsub was calculated based on the AMB substrate, while SASApkt was based on residues H72, D74, W77, D196, R220, D319, H446, D447, W660, and R82 from the active site. All calculations were done with cpptraj^56^ within the suite of AmberTools23.

## Results and Discussion

First, we explored the conformational free energy landscape (FEL) of the mannose ring −1 of the model substrate α-d-mannopyranosyl-(1 → 6)-β-d-mannopyranose (α-mannobiose, AMB) at the active site of wild-type hLAMAN (Figure 2). We docked the substrate by structural superimposition of AMB in complex with the hGH125 1,6-α-mannosidase variant (PDB id. 5M7I,^25^Section S1). The initial geometry of the enzyme–substrate (ES) complex was refined by using classical MD simulation calculations. We defined as collective variables for QM/MM metadynamics the periodic Cremer–Pople puckering coordinates ϕ (between 0 and 2π) and the nonperiodic angle θ (between 0 and π, Figure 2A).^57−59^ The substrate, the two catalytic residues D196 and D319, the metal center, and the side chain of the residues at its coordination sphere were treated at the density functional theory (DFT) level using the standard DFT functional PBE for the simulation of GH-based catalysis^39,40^ with the Ahlrichs double-ζ basis sets (def2-SVP).^41^ The rest of the system was treated with the ff19SB force field for the protein residues and GLYCAM06^27^ for the sugar moieties (see Methods section).

Initial attempts using the semiempirical tight-binding density functional theory (DFTB3)^60^ for the QM region showed an energy minimum around an *E*_5_ conformation separated by 1–2 kcal mol^–1^ with the conformations ^4^*H*_5_ and ^*O*^*H*_5_ (see Figure S3 and Section S1).^61^ Other explored conformational regions show a local minimum in the ^1^*S*_3_ space being 3 kcal mol^–1^ less favorable than the *E*_5_. The expected ^O^*S*_2_ conformation reported for retaining α-mannosidases of the GH38 family (e.g., hGMII)^4^ is not energetically favorable in our case. Although the substrate presents a ^*O*^*S*_2_ conformation in our initial geometry, we obtained again a local minimum at the *E*_5_ conformation using PBE/def2-SVP (Figure 2B). This minimum is 10 kcal mol^–1^ away from the canonical conformation ^*O*^*S*_2_(62) and 5 kcal mol^–1^ away from the second minimum on the free energy landscape (between conformations ^1^*S*_3_ and ^1,4^*B*). In our understanding, the differences obtained in hLAMAN in comparison with hGMII for the puckering of the substrate may arise due to the use of atomic orbital basis set functions (no plane waves) and the nature of the active site due to the presence of a metal center. These results are consistent with previous findings in Golgi α-mannosidase II (GH92) of one of the authors.^63^

We analyzed the puckering for the same substrate but at the active site of the defective D74E variant, a dormant mutant with only 11% of the wild-type activity (Figure 2C). In principle, the change from aspartic acid to glutamic acid only incorporates one extra CH_2_ into the side chain of this residue located at the Zn^2+^-coordination sphere. However, we see that the puckering of the substrate shows now a very different conformational energy profile than when bound to the wild-type counterpart. The *E*_5_ conformation is energetically unfavorable toward the ^1^*S*_3_ by more than 14 kcal mol^–1^. Upon visual inspection of both ES complexes, we observed that while the distances between the nucleophilic oxygen and the anomeric carbon in the substrate are similar for both variants (distance O_*n*_–C1′, Table 1), the angle for the nucleophilic attack O_*n*_–C1′–O_*ring*_ as well as the interaction with near residues (i.e., R220) are affected (Figure 2). These findings for the change of the puckering in the substrate as consequence of an amino acid exchange at the active site can be observed in experimental structures of other members of the GH38 family.^64,65^ A similar case has also been reported in *Arabidopsis thaliana* cell-wall invertase, where the substrate–enzyme interactions are affected by mutations of residue D239.^66^ As an exercise, we analyzed the Cremer–Pople puckering of the experimental conformation of different mannose-based substrates bound to the fruit fly GMII defective variant D304 (Table S2). When this nucleophilic residue is changed to alanine, the substrate populates the nonreactive conformation ^4^*C*_1_.

**Table 1:** Collective Variables, Distances, and Bonds for the ES Complex, ETS Complex, and GEI of Wild-Type and D74E hLAMAN Variants

variable	CV_1_ (Å)	CV_2_ (Å)	C1′–O_*g*_ (Å)	O_*n*_–C1′ (Å)	O_*a*_–H_*a*_ (Å)	H_*a*_–O_*g*_ (Å)
wild-type ES	–0.16 ± 0.11	–1.66 ± 0.11	1.48 ± 0.04	3.05 ± 0.13	1.02 ± 0.03	1.65 ± 0.09
wild-type ETS	0.96 ± 0.11	0.33 ± 0.09	2.36 ± 0.10	2.35 ± 0.09	1.50 ± 0.12	1.05 ± 0.03
wild-type GEI	1.31 ± 0.11	1.521 ± 0.11	3.42 ± 0.11	1.49 ± 0.06	1.63 ± 0.12	1.02 ± 0.04
D74E ES	–0.03 ± 0.11	–1.52 ± 0.11	1.44 ± 0.04	3.05 ± 0.20	1.25 ± 0.23	1.62 ± 0.23
D74E ETS	1.11 ± 0.11	–0.59 ± 0.11	2.09 ± 0.25	2.59 ± 0.11	1.61 ± 0.13	1.08 ± 0.04
D74E GEI	1.30 ± 0.11	1.33 ± 0.11	3.17 ± 0.18	1.48 ± 0.06	1.90 ± 0.16	1.00 ± 0.04

**Scheme 1:**
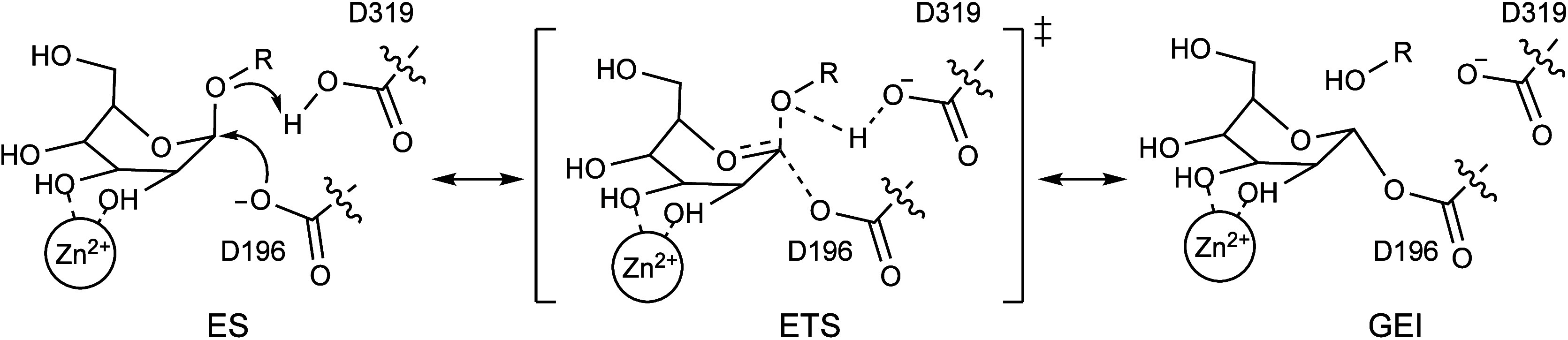
Proposed Enzyme-Based Reaction Mechanism for the Glycosylation Step of the Cleavage of an α(1 → 6) Bond between Two Mannose Rings in the Substrate AMB (Enzyme–Substrate Complex, ES) to Generate the Glycosyl–Enzyme Intermediate (GEI) though the Corresponding Transition State (ETS Complex) R represents the mannose ring +1 of AMB.

Next, we simulated the first step of the reaction mechanism of the hydrolysis of AMB for both wild-type and D74E enzymes to compute the impact of this mutation on the reaction energy barrier. This is the accepted rate-limiting step of the hydrolytic mechanism within the GH38 family, and it implies the formation of the covalent glycosyl–enzyme intermediate (GEI in Scheme 1).^62,67,68^ In hLAMAN, D196 acts as the nucleophile, and D319 acts as the general acid/base catalyst. As a retaining glycosidase, the product released after hLAMAN catalysis keeps the absolute configuration on the anomeric carbon of the −1 mannose ring.^69,70^ We defined two collective variables for the reaction mechanism (Figure 3A): CV_1_, composed by the difference between the distance of the center-of-mass (COM) of the carboxylic oxygen atoms in D319 and the proton H_*a*_ on the same residue (*d*_1_) and the distance between the former H_*a*_ atom and the glycosidic oxygen O_*g*_ in the substrate (*d*_2_); and CV_2_, defined as the difference between the distance of the O_*g*_ oxygen (+1 ring) and the former C1′ in the −1 ring (*d*_3_) and the distance between the anomeric carbon C1′ and the COM of the carboxylate oxygen atoms in D196 (*d*_4_). The values of CV_1_, CV_2_, and the former distances for the ES complex, enzyme–transition state (ETS) complex, and GEI are summarized in Table 1.

Figure 3B shows the 2D free energy landscape of the catalyzed reaction by wild-type hLAMAN. A reaction free energy of activation ΔG^‡^ of 19.8 kcal mol^–1^ is computed from the minimum free energy pathway (MFEP, black line), a value that is in agreement with the experimental value of ca. 20 kcal mol^–1^ for the cleavage of α(1 → 6)-linked mannobioses.^71^ In our calculations, the reaction is slightly exergonic with Δ*G* = −2.99 kcal mol^–1^ between the ES complex and GEI. Looking in detail at the evolution of the relevant distances along the catalyzed reaction mechanism, the leaving group leaves earlier than the oxygen O_*g*_ of D196 attacks the anomeric carbon C1′ (see *C*1′–O_*g*_ vs O_*n*_–C1′ at the ETS, Figure 3C). Therefore, the reaction may follow a dissociative nucleophilic addition. Regarding the puckering evolution along the reaction, the substrate moves from the *E*_5_ region to an ETS with *E*_5_/*B*_2,5_ character, and it ends up in a ^1^*S*_5_ conformation in the enzyme intermediate (Figure 3D and Table S4). The puckering of the ETS complex is in good agreement with other reported ETS complexes within the retaining α-mannosidase families (i.e., GH38, GH76, GH92, and GH125).^4,62,72,73^

**Figure 4:**
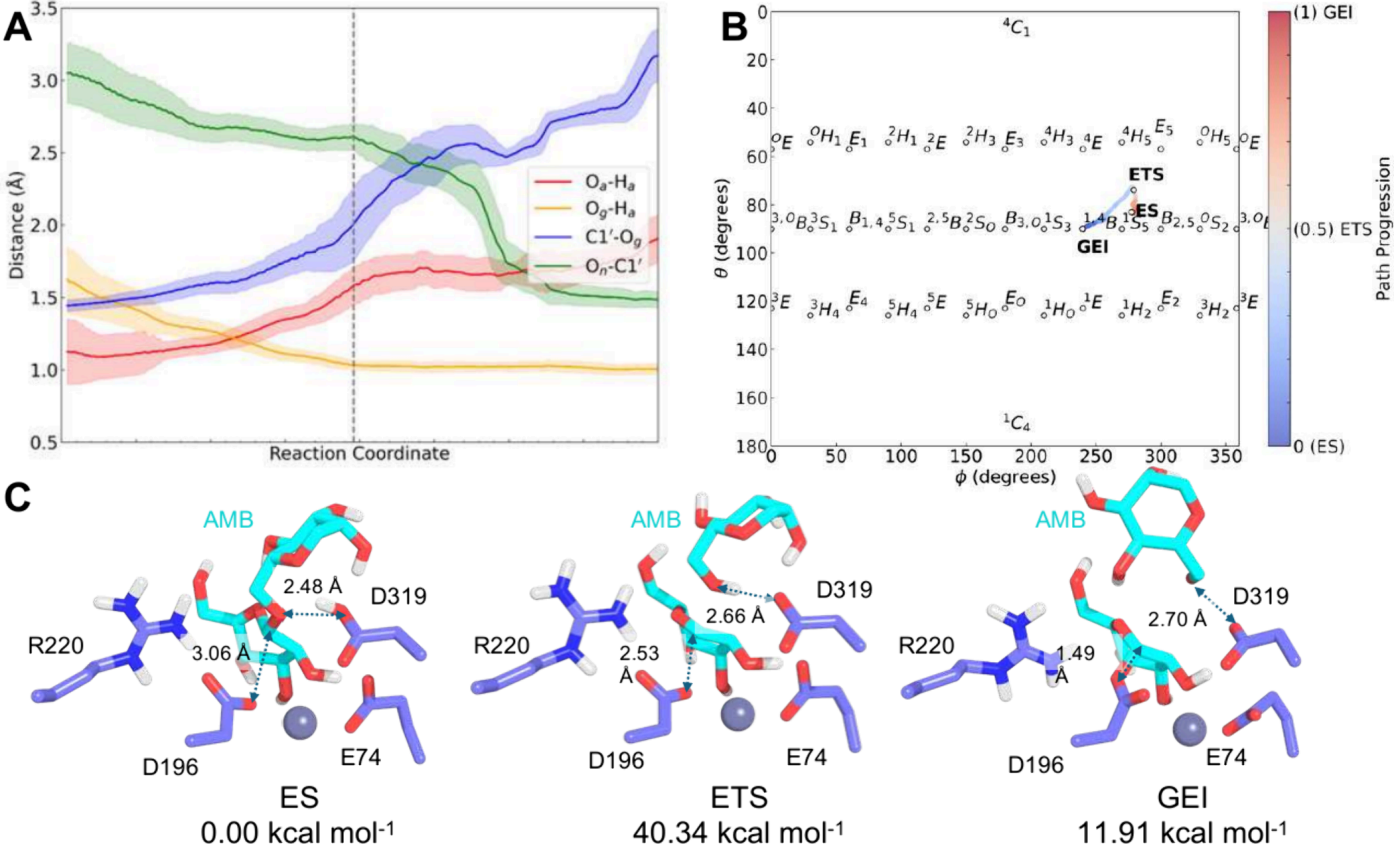
(A) Evolution of catalytic distances (Å, running averages over 0.2 Å data points) along the D74E hLAMAN MFEP: O_*a*_–H_*a*_ (red), H_*a*_–O_*g*_ (orange), C1′–O_*g*_ (blue), and O_*n*_–C1′ (green). The ETS is highlighted with a dotted line. (B) Puckering evolution along the reaction. (C) Representative average structure along the MFEP for the ES complex, ETS complex, and GEI of D74E hLAMAN.

Subsequently, we explored the reaction mechanism for the inactive mutant D74E following the same protocol (Figure 4 and Figure S5). The reaction followed a dissociative mechanism (Figure 4A). Our reaction started from the minimum of energy of the puckering analysis (^1^*S*_3_), and we observed a boat conformation ^1,4^*B* in the ring −1 of AMB (Figure 4B and Figure S5, Table 1). From there, the system evolved through the corresponding ETS but, unexpectedly, not by following the shortest path to the ^1^*S*_5_ conformation, but rather the same path as for the wild-type *E*_5_/*B*_2,5_. As an outcome, the D74E hLAMAN variant presents an energy barrier of 40 kcal mol^–1^, twice as much as the one computed for the wild-type counterpart. Moreover, the reaction is now endergonic by about 12 kcal mol^–1^ (Figure 4C). As expected, calculations of this catalytic step with DFTB3 by the wild-type and D74E hLAMAN variants underestimate the energy barrier for this enzyme variant (Figures S6 and S7).^61^ Regarding the conformational pathway, similar geometries are observed for both enzymes. Overall, by comparing the energy profiles for the reaction as well as the puckering population of the substrate along the reaction for both wild-type and D74E variants, we obtained valuable information about how differences in the substrate puckering ring affect the enzyme activity in hLAMAN.

**Figure 5:**
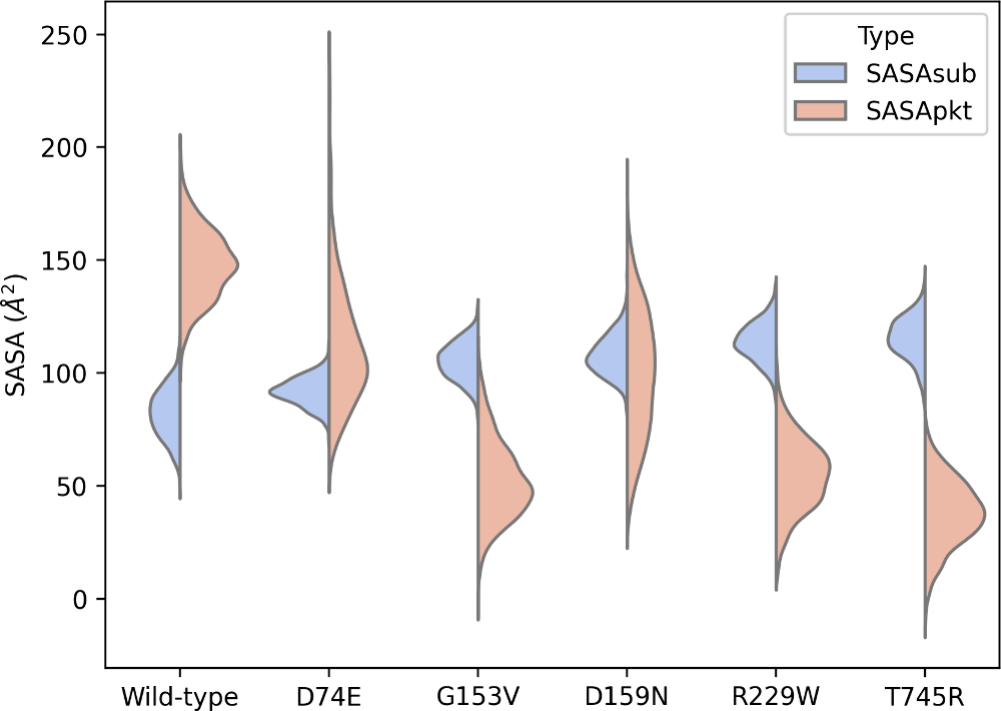
Violin plot for the SASA (Å^2^) of the substrate (SASAsub) and the active site (SASApkt) of the wild-type and the five studied hLAMAN variants.

Since most of the amino acid changes in hLAMAN resulting from the reported missense mutations are located more than 10 Å away from the residues at the active site, we were interested to see if we could detect substrate puckering alterations by placing remote single-point mutations onto the hLAMAN scaffold. We selected four reported enzyme variants G153V, D159N, R229W, and T745R, where the enzyme can fold but presents a reduced enzymatic activity (Table S1), and carried out the puckering conformational analysis as done before (Figure S8). We found significant differences in their energy minima and their Michaelis complex geometries when compared with the wild-type counterpart (Table 2). On the one hand, the length of the glycosidic bond and the distance between the nucleophilic oxygen and the anomeric carbon at the ES complex minima (*d*_3_ and *d*_4_, respectively, in Figure 3A) are both affected (Figures S9 and S10). In the D74E and D159N variants, with ^1^*S*_3_ and ^1,4^*B* AMB conformations, respectively, the glycosidic bond is shortened, and the nucleophilic oxygen moves away from the electrophile. In the case of the two variants G153V and T745R, the nucleophilic attack distance is enlarged. In the former variant, the substrate adopts a ^1,4^*B* conformation; in the latter, it adopts a TS-like conformation (^4^*H*_5_/^1^*S*_5_). As shown, the defective enzyme variants populate a variety of nonreactive conformations. To quantify these populations, we calculated for each of the variants the ratio to free energies of reactive and nonreactive explored conformations (Δ*G*_*r*/*nr*_).^54^ For α-mannosidases, the reactive conformations of the substrate are located on the space between ^4^*C*_1_ and the transition state conformational space in the 2D representation of the puckering coordinates (φ,θ). The transition state conformational space was computed in a previous work based on the screening of the puckering of several transition state mimic inhibitors in glycoside hydrolases.^54^ Nonreactive conformations are those located below the space of the transition states and ^1^*C*_4_. As an example, *E*_5_ is located between ^4^*C*_1_ and the transition state conformational space, and it is considered reactive. On the contrary, ^1^*S*_3_ is located on the southern part and is considered nonreactive. The more negative the Δ*G*_*r*/*nr*_, the larger the stability of reactive conformations. As shown in Table 2, the majority of variants populate nonreactive conformations to a greater extent than the wild-type enzyme, with positive Δ*G*_*r*/*nr*_ values (D74E, D159N) or less negative values (R229W, T745R, G153V). On the other hand, we also found differences in the ratio between the solvent-accessible surface area of the substrate (SASAsub) and the one of the active-site pocket (SASApkt) by computing the substrate-positioning index (SPI)^55^ (Figure 5 and Table 2). SPI refers to the ratio of SASAsub and SASApkt. All enzyme variants with a remote modification present a larger value for SASApkt when compared to the wild-type and, therefore, larger SPI values.^74^ In particular, R229W and T745R are the ones with the highest SPI values. Although we have only explored five enzyme variants, our data indicate the use of SPI as a potential descriptor not only for the mutation’s influence on activation-free energies in enzyme-based reactions^74^ but also to explore pathological mutations affecting enzyme activity.^55,75^

**Table 2:** Analysis of the 2D Conformational Free Energy Surface for the Puckering of the −1 Ring of AMB at the Active Site of Wild-Type hLAMAN and D74E, G153V, D159N, R229W, and T745R Variants

variant	conformation^a^	Δ*G*_*r*/*nr*_^b^	substrate-positioning index
wild-type	*E*_5_	–4	0.55
D74E	^1^*S*_3_	+10	0.77
G153V	*E*_5_/*B*_2,5_/^1,4^*B*	0	2.03
D159N	^1,4^*B*	+4	1.09
R229W	*E*_5_/*B*_2,5_	–2	2.09
T745R	^4^*H*_5_/^1^*S*_5_	–1	3.08

a Global energy minimum.

b kcal mol^–1^.

## Conclusion

Overall, we have shown in this work for the first time that one of the mechanisms of how remote pathogenic mutations reduce the hLAMAN enzyme activity is by changing the puckering of the substrate in the ES complex toward nonreactive conformations. This way, the glycosidic bond for cleavage and/or the nucleophilic attack are both less activated. This puckering change is a consequence of the reduction of the SASA of the active-site pocket changes. In addition to that, the study of the key step of the catalytic reaction mechanism for both wild-type and the orthosteric defective hLAMAN D74E variant shows how in the latter variant, where the substrate populates a different puckering, the energy barrier of the reaction is significantly increased. We have made use of conformational ensembles derived from extensive QM/MM metadynamics, which usually have a high computational cost. Nevertheless, its combination with deep-learning-based models offers a powerful way to understand how point mutations affect the enzyme activity in LSDs. Indeed, these approaches can improve treatment selection and may help guide the design and development of new therapies.

## Data Availability

Data for the simulation of wild-type and D74E, G153V, D159N, R229W, and T745R human lysosomal α-mannosidase variants are publicly available (10.5281/zenodo.14251812). A folder has been created for each of the enzyme variants including the following: both top and crd files for Amber MD simulations, metadynamics data for the exploration of the puckering of the mannose ring −1 in AMB (all enzyme variants), and metadynamics data for the first step for the enzymatic cleavage of the glycosidic bond in AMB (wild-type, D74E).

## Supporting Information Available

All methods are described in the different sections, and additional figures and tables are provided (PDF)
