# Supplementary material for: Digging out the Molecular Connections between the Catalytic Mechanism of Human Lysosomal α-Mannosidase and Its Pathophysiology
Source: J Chem Inf Model. 2025 Feb 20;65(5):2650–9. doi: 10.1021/acs.jcim.4c02229 (PMC11898060; doi:10.1021/acs.jcim.4c02229)
Supplement: Supplementary file 1 — ci4c02229_si_001.pdf [file ci4c02229_si_001.pdf]

Supporting Information: Digging out the molecular connections between the catalytic mechanism of human lysosomal alpha-mannosidase and its pathophysiology

Bruno Di Geronimo,[†,‡] Špela Mandl,[†] Santiago Alonso-Gil,[*,¶,§] Bojan Žagrović,[¶,§] Gilber Reibnegger,[†] Christoph Nusshold,[†] and Pedro A. Sánchez-Murcia[*,†,∥]

†*Laboratory of Computer-Aided Molecular Design, Division of Medicinal Chemistry, Otto-Loewi Research Center, Medical University of Graz, Neue Stiftingtalstr. 6/III, A-8010 Graz, Austria*

‡*Present address: School of Chemistry and Biochemistry, Georgia Institute of Technology, Atlanta, Georgia, USA*

¶*Max Perutz Labs, Vienna Biocenter Campus (VBC), Campus Vienna Biocenter 5, 1030, Vienna, Austria*

§*Department of Structural and Computational Biology, Vienna BioCenter University of Vienna Campus-Vienna-Biocenter 5, A-1030 Vienna, Austria*

∥*BioTechMed-Graz, Mozartgasse 12/II, A-8010 Graz, Austria*

E-mail: santiago.alonso.gil@univie.ac.at; pedro.murcia@medunigraz.at

Supplementary Figures

S1 Positions explored in this work. Their location on the structure of hLAMAN are highlighted as spheres on the left. On the right, the local environment of these positions is shown. 4

S2 QM region used in this work. 4

S3 Puckering screening of mannose ring -1 of AMB at the active site of wild-type hLAMAN variant using DFTB3/ff19SB. 6

S4 Puckering screening of mannose ring -1 of AMB at the active site of D74E hLAMAN variant using DFTB3/ff19SB. 7

S5 2D free energy surface (kcal mol^{-1}) for the first step of the hydrolysis of AMB at the active site of D74E hLAMAN using PBE/def2-SVP. The MFEP is shown with a black line and EP for the glycosyl-enzyme intermediate (GEI). 8

S6 (A) Collective variable definition used in the QM/MM metadynamics. (B) 2D free energy surface (kcal mol^{-1}) for the first step of the hydrolysis of AMB at the active site of wild-type hLAMAN using DFTB3/ff19SB. The MFEP is shown with a dotted black line. (C) Detail of the geometries of ES complex, ETS complex, and GEI. 9

S7 (A) 2D free energy surface (kcal mol^{1}) for the first step of the cleavage reaction of AMB at the active site of D74E hLAMAN variants using DFTB3/ff19SB. The MFEP is shown with a dotted black line. (B) Structural comparison between both ES complexes in wild-type and D74E hLAMAN. 10

S8 Conformational 2D free energy surface (kcal mol^{-1}) for the puckering of the mannose ring -1 of the AMB at the active site of G153V, D159N, R229T, and T745R hLAMAN variants calculated using PBE/def2-SVP. 11

S9 Surface of the average value of d_3 (Å) along the conformational 2D-FES of wild-type, D74E, G153V, D159N, R229W, and T745R hLAMAN variants. . 12

S10 Surface of the average value of d_4 (Å) along the conformational 2D-FES of wild-type, D74E, G153V, D159N, R229W, and T745R hLAMAN variants. . 12

Supplementary Tables

S1 17 hLAMAN defective enzyme variants paired with its respective experimental data:[S1] percentage activity, distance to the catalytic Zn^{2+} atom, chain, located motive position, and if there is contact with other chains. 13
S2 Puckering analysis of α-D-mannopyranose and derivatives from *D. melanogaster* GH38 α-mannosidase (UniProt id. Q24451) deposited in the PDB.[S2–S4] . . . 13
S3 Cremer-Pople angles ϕ and θ along the reaction pathway for wild-type and D74E variants. 14
S4 Calculated SASAsub, SASApkt (Å^2) and substrate-positioning index (SPI) values along the QM/MM metadynamics trajectories. 14
S5 Distance difference (Δd, Å) between α-carbon atoms of residues H72, D74, D196, D319, H445, H446, and D447 in the equilibrated structures of the G153V, D159N, R229W, and T745R hLAMAN enzyme variants and equilibrated structure of wild-type hLAMAN after structural superimposition. . . 14

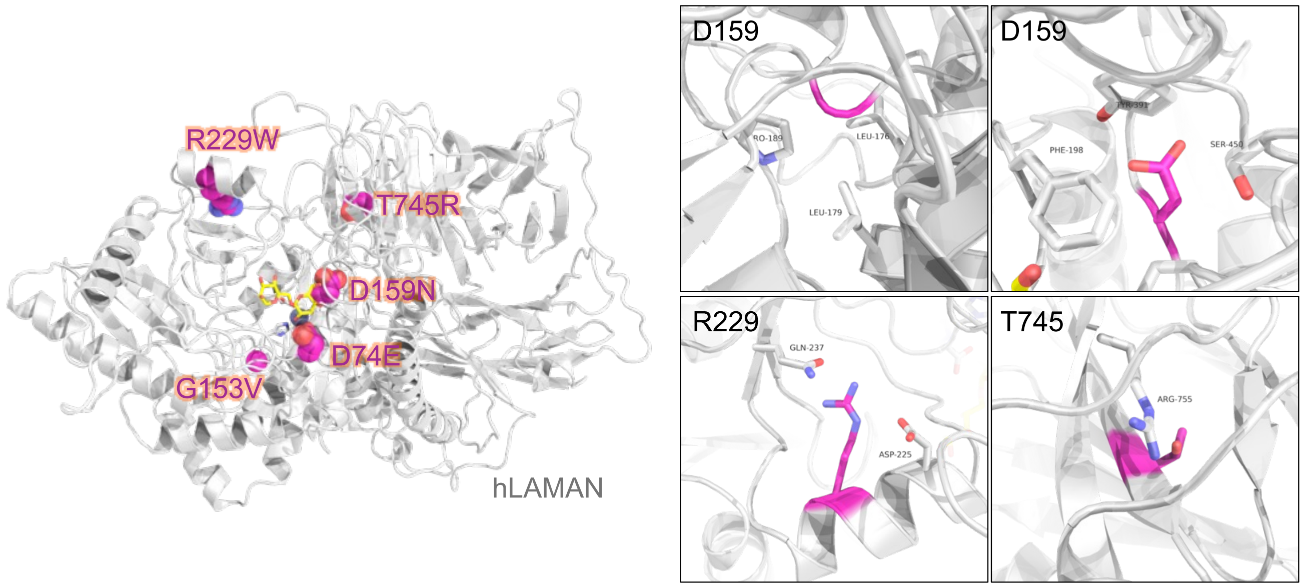

Figure S1 : Positions explored in this work. Their location on the structure of hLAMAN are highlighted as spheres on the left. On the right, the local environment of these positions is shown.

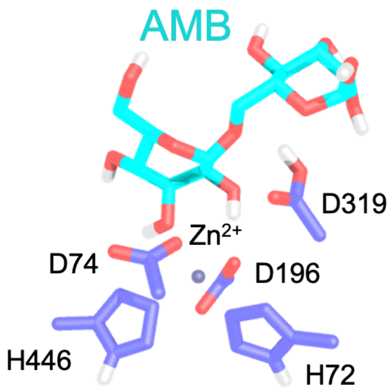

Figure S2 : QM region used in this work.

Section S1: Preliminary simulations using DFTB3

We explored initially the puckering of the substrate at the active site of both wild-type and D74E hLAMAN variants following the protocol reported in Section S2 but treating the QM

region with DFTB3 (Figure S??).[S5] We made use of the DFTB3 version implemented within Amber20.[S6] For the puckering analysis, the sumulations were stopped after having added 2,000 (wild-type) and 16,000 (D74E) Gaussian terms for 120 and 96 ps, respectively.
For the reaction mechanism, a he total number of added Gaussian terms was 2,836 (wild-type) and 4,968 (D74E variant) with a total simulated time of 170 ps and 296 ps, respectively, until the metadynamics convergences.

- **Substrate puckering**. We found in wild-type hLAMAN that the minimum of energy is located around an E_5 conformation separated by 1-2 kcal mol^{-1} with the conformations $^4H_5$ and $^OH_5$ (Figure S3). Other explored conformational regions show a local minimum in the $^1S_3$ space being 3 kcal mol^{-1} less favorable than the E_5. Regarding the D74E hLAMAN variant, we found out that the puckering of the reactive ring in AMB shows a different conformational energy profile than when bound to the wild-type counterpart (Figure S4). Now, the E_5 conformation is energetically unfavorable towards a $B_{2,5}$ conformation.

- **Reaction mechanism**. In Figure S5 A are shown our definition of CVs and the 2D-free energy landscape of the catalyzed reaction by the wild-type enzyme. The black line specifies the minimum free energy pathway (MFEP). The ES complex evolves to the GEI via a transition state (ETS) with a very low QM/MM energy barrier ΔG of 7.2 kcal mol^{-1} (Figure S5 B). Clearly, DFTB3 overestimates the energy barrier when compared to PBE. Recalculation of this energy barrier with Nudged Elastic Band method (NEB) with GFN2-xTB delivers a value of 19.8 kcal mol^{-1}. We simulated the reaction mechanism for the same substrate AMB. We obtained a DFTB3 energy barrier of 12.3 kcal mol^{-1}, 5.1 kcal mol^{-1} (+75 %) larger than for the wild-type (Figure 3B). The height of the barrier increases up to a value of 37.3 kcal mol^{-1} via the NEB method (GFN2-xTB/ff19SB). A detail of the geometries for the ES complex, ETS complex and GEI can be seen in Figure S5 C. Further details are included in our preprint in ChemRxiv.[S7]

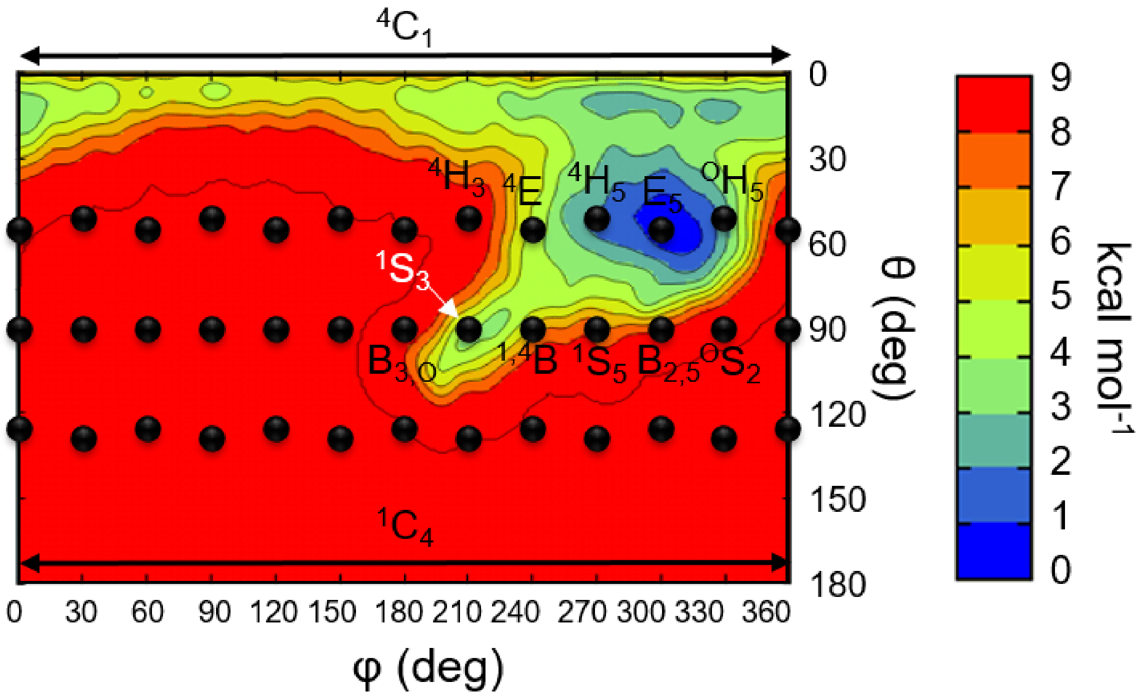

Figure S3 : Puckering screening of mannose ring -1 of AMB at the active site of wild-type hLAMAN variant using DFTB3/ff19SB.

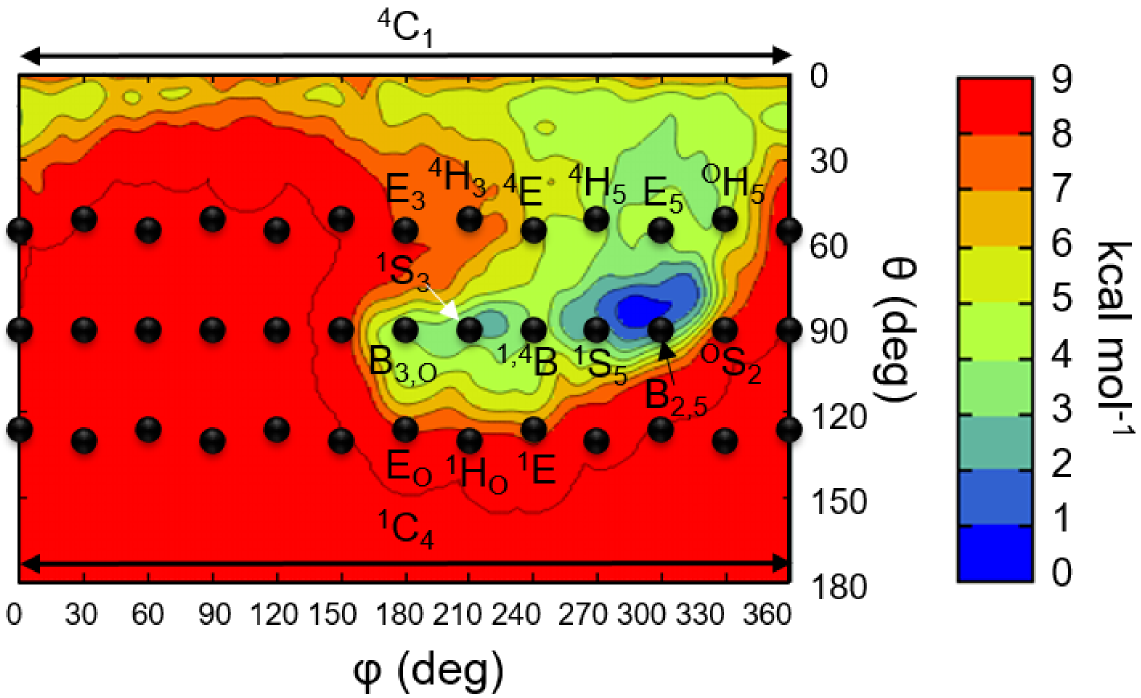

Figure S4 : Puckering screening of mannose ring -1 of AMB at the active site of D74E hLAMAN variant using DFTB3/ff19SB.

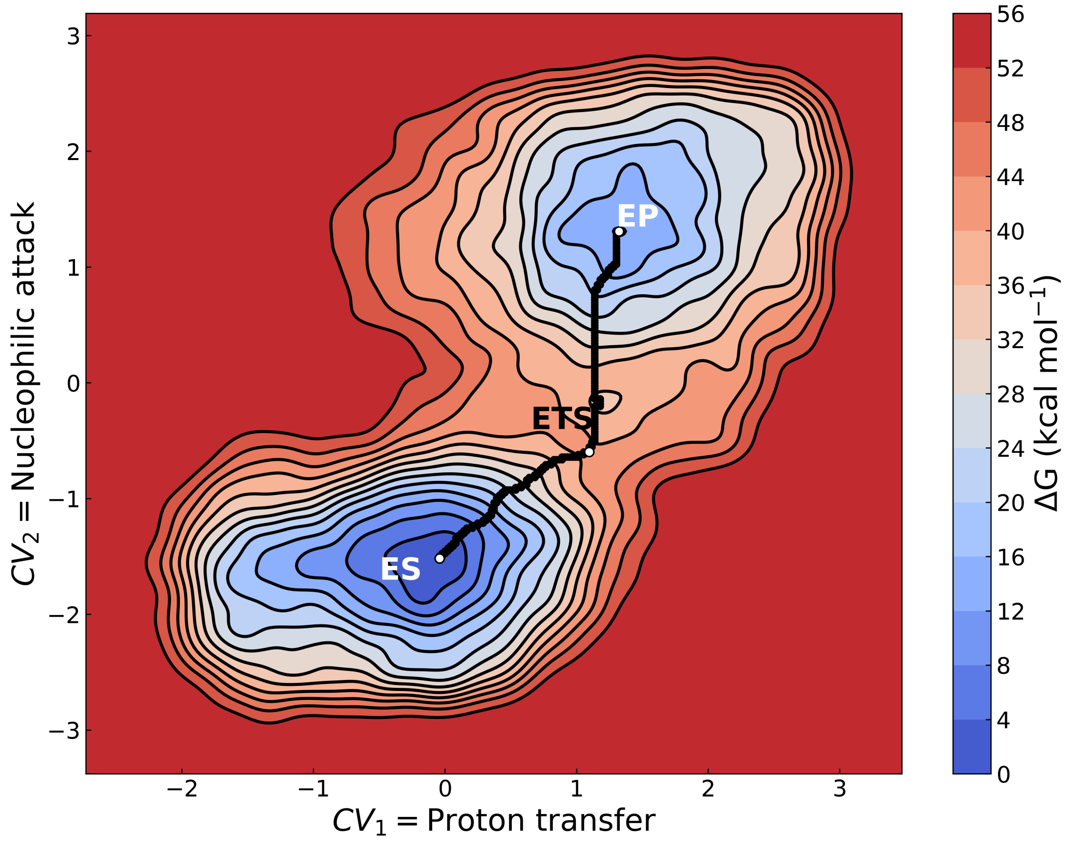

Figure S5 : 2D free energy surface (kcal mol^{-1}) for the first step of the hydrolysis of AMB at the active site of D74E hLAMAN using PBE/def2-SVP. The MFEP is shown with a black line and EP for the glycosyl-enzyme intermediate (GEI).

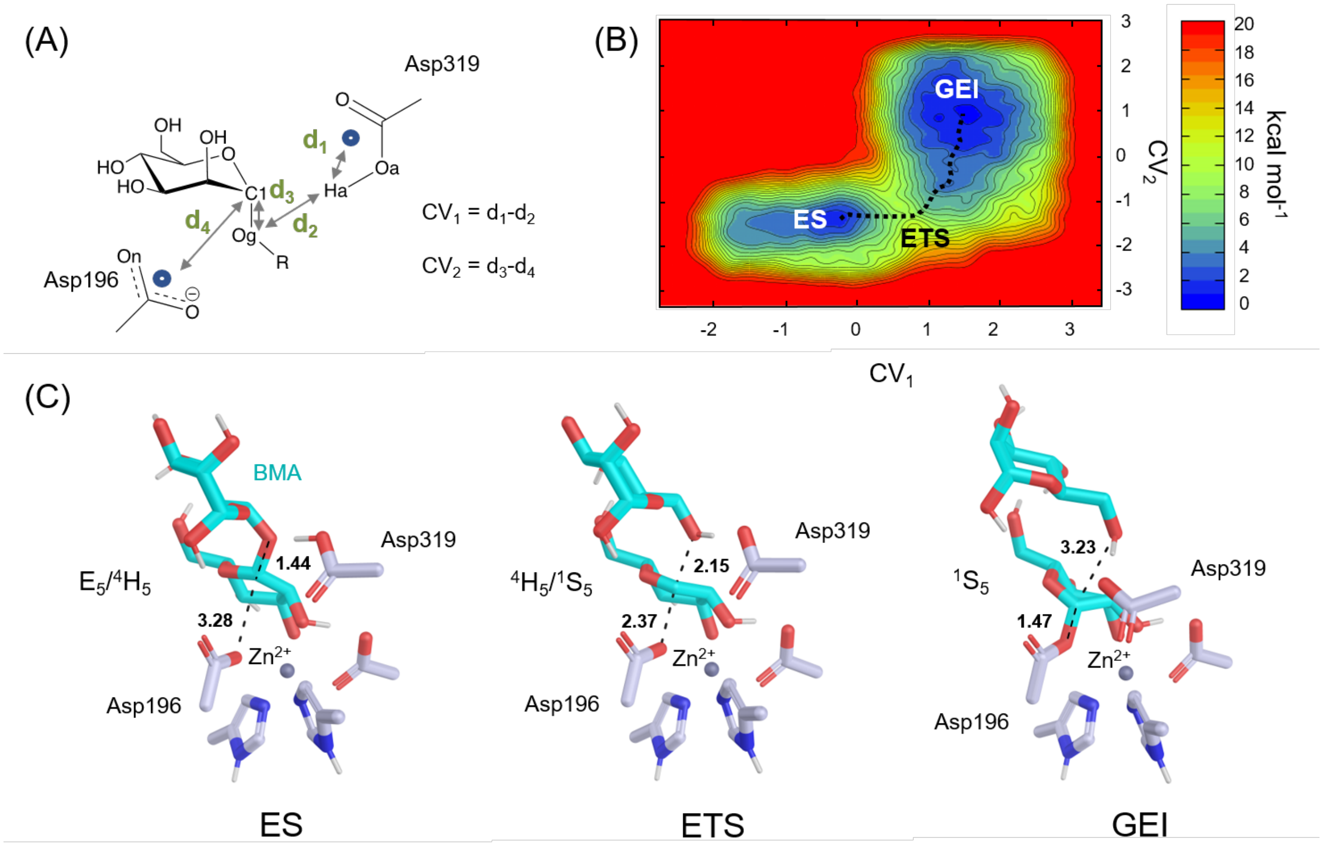

Figure S6 : (A) Collective variable definition used in the QM/MM metadynamics. (B) 2D free energy surface (kcal mol^{-1}) for the first step of the hydrolysis of AMB at the active site of wild-type hLAMAN using DFTB3/ff19SB. The MFEP is shown with a dotted black line. (C) Detail of the geometries of ES complex, ETS complex, and GEI.

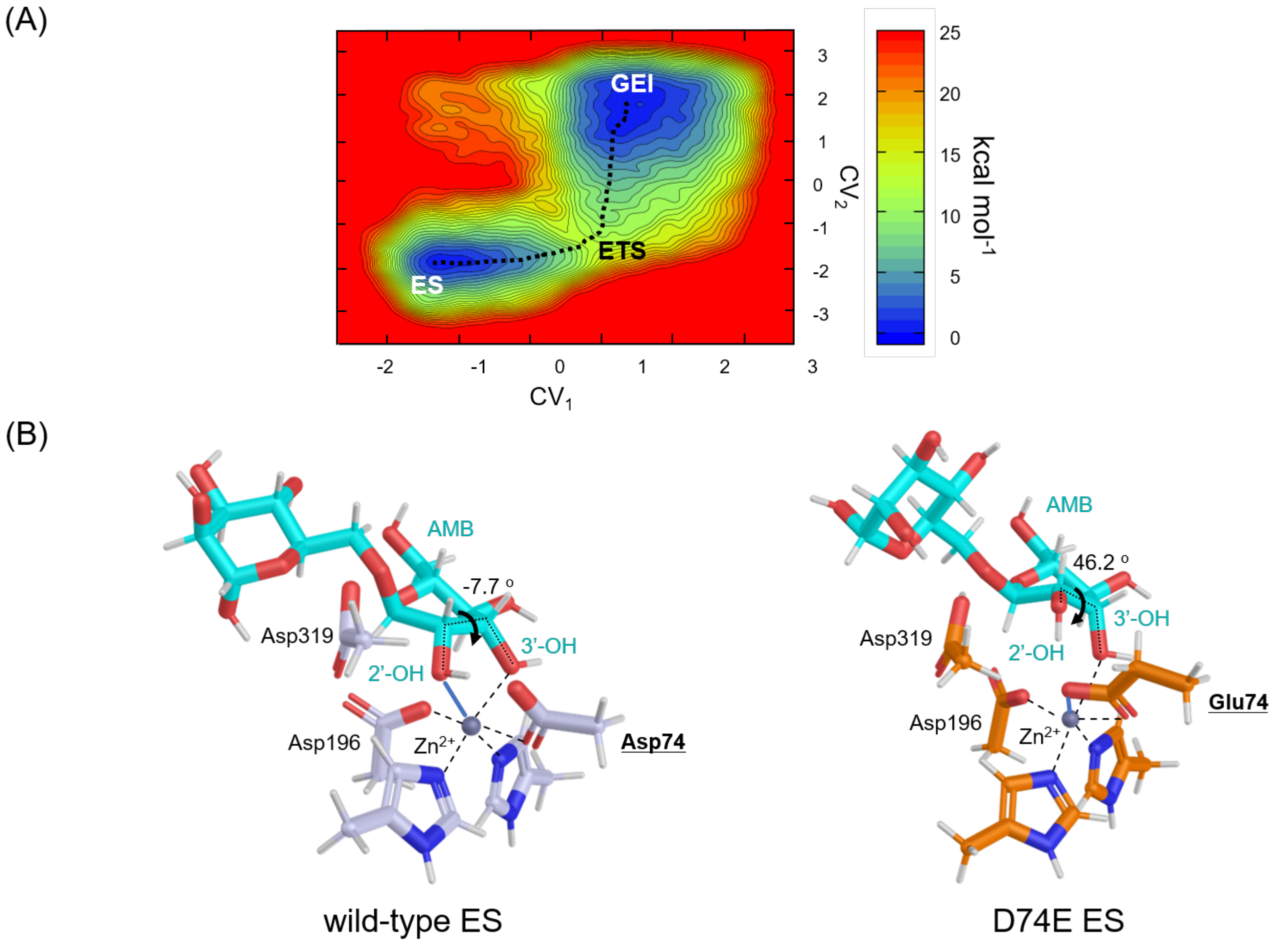

Figure S7 : (A) 2D free energy surface (kcal mol^1) for the first step of the cleavage reaction of AMB at the active site of D74E hLAMAN variants using DFTB3/ff19SB. The MFEP is shown with a dotted black line. (B) Structural comparison between both ES complexes in wild-type and D74E hLAMAN.

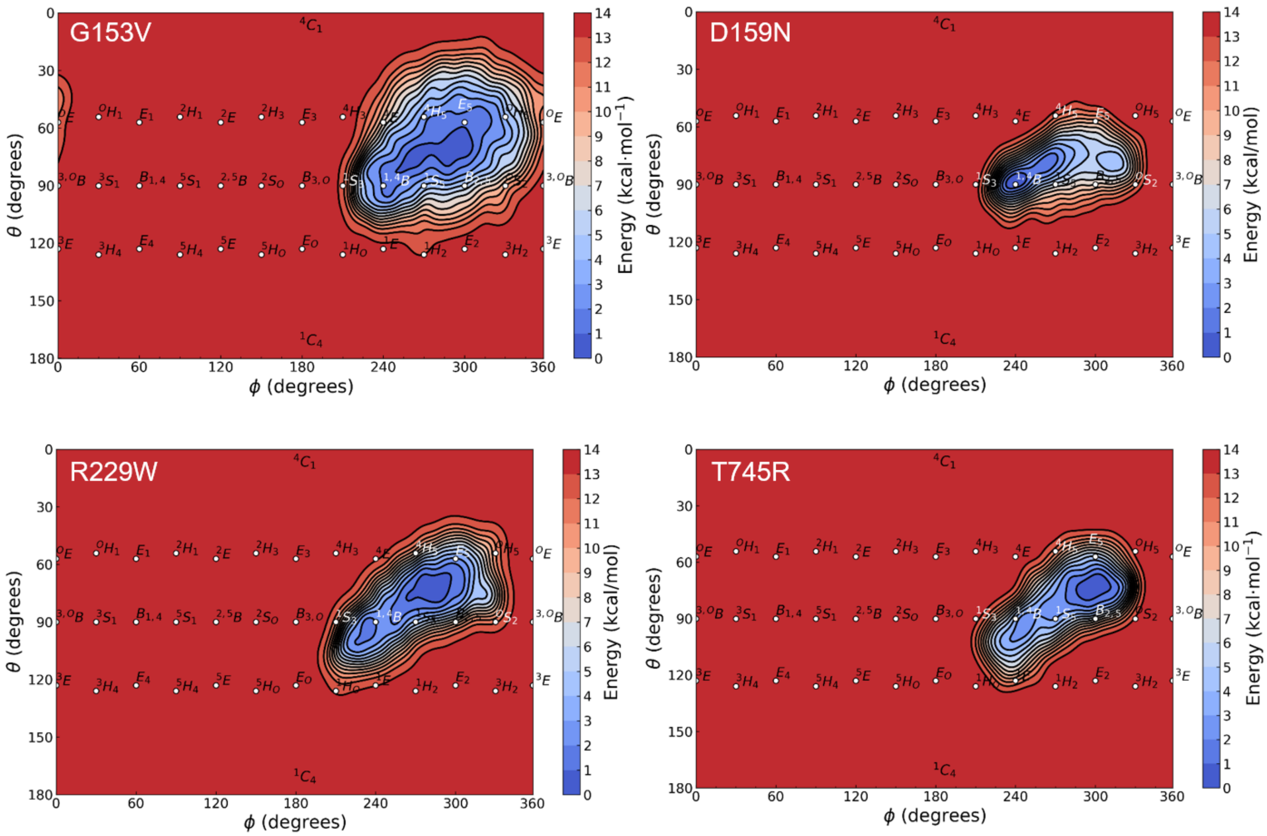

Figure S8 : Conformational 2D free energy surface (kcal mol^{-1}) for the puckering of the mannose ring -1 of the AMB at the active site of G153V, D159N, R229T, and T745R hLAMAN variants calculated using PBE/def2-SVP.

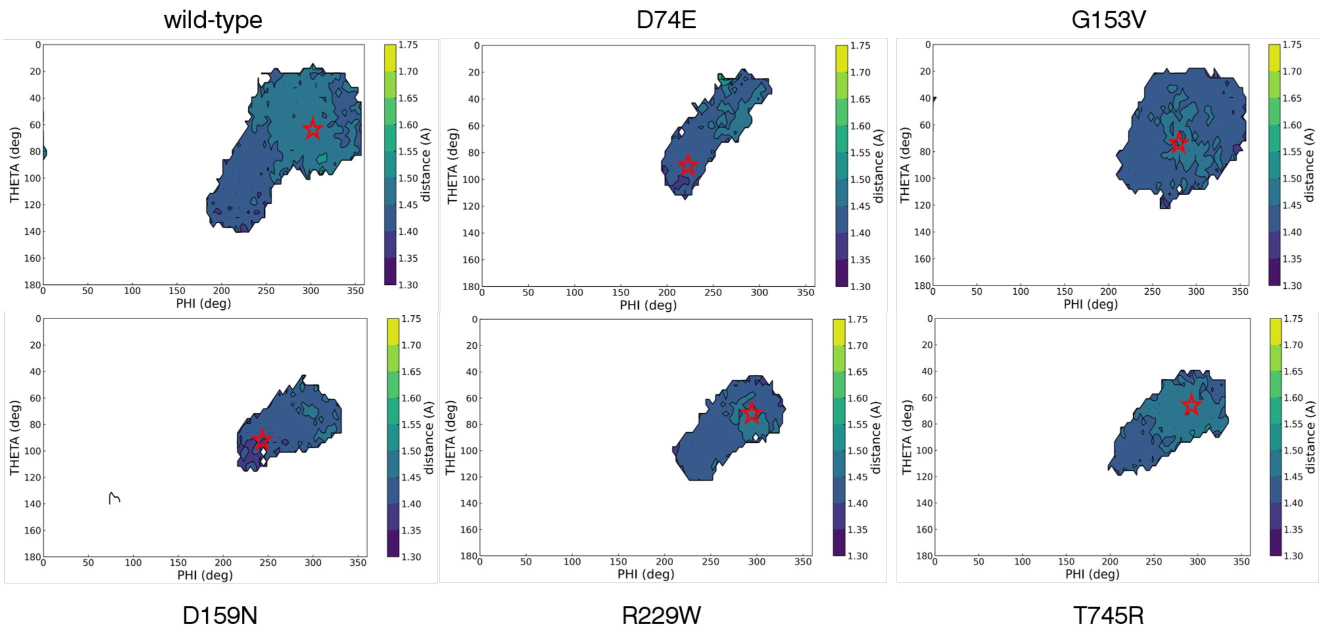

Figure S9 : Surface of the average value of d_3 (Å) along the conformational 2D-FES of wild-type, D74E, G153V, D159N, R229W, and T745R hLAMAN variants.

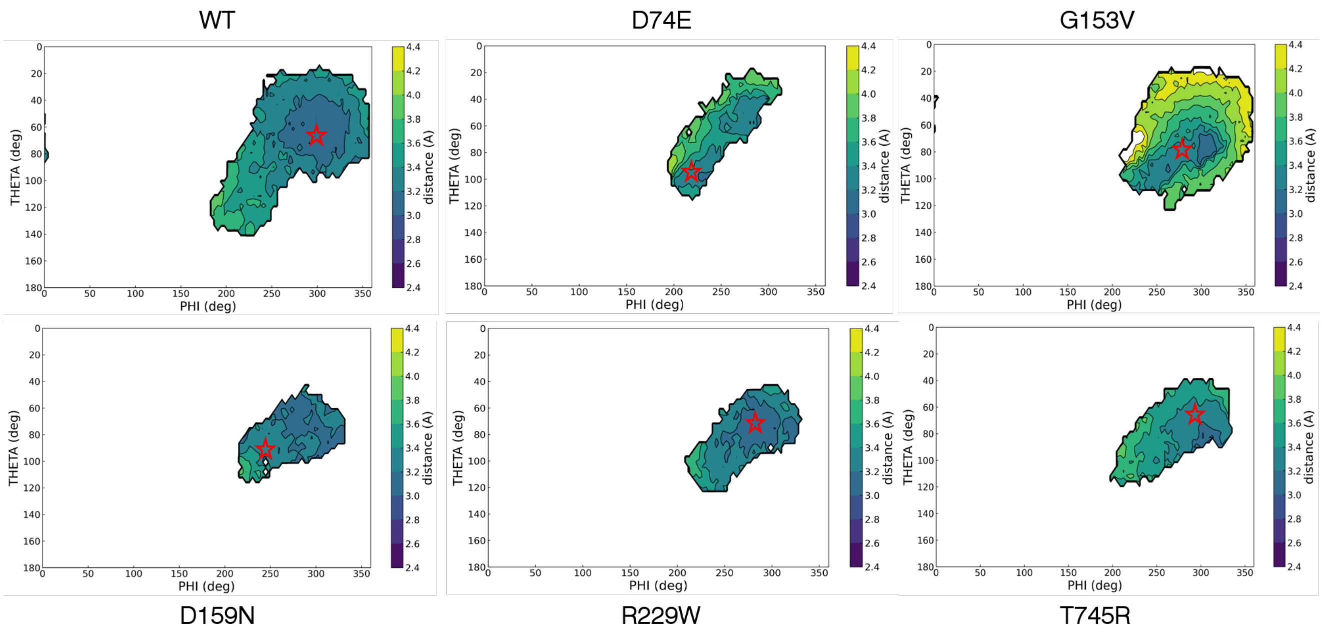

Figure S10 : Surface of the average value of d_4 (Å) along the conformational 2D-FES of wild-type, D74E, G153V, D159N, R229W, and T745R hLAMAN variants.

Table S1 : 17 hLAMAN defective enzyme variants paired with its respective experimental data:[S1] percentage activity, distance to the catalytic Zn^{2+} atom, chain, located motive position, and if there is contact with other chains.

Mutant	Activity (%)**	Distance	Chain***	Position	Contact
H72L	17	6.5	A	Active Site	-
D74E	11	5.0	A	Active Site	-
Y99H	9	18.5	A	α-helix 3	-
D102N	15	21.0	A	α-helix 3	-
G153V	19	14.9	A	loop	-
D159N	10	9.7	A	loop	B,C
H200L	25	13.9	A	loop	-
H200N	53	13.9	A	loop	-
R229W	30	23.7	A	α-helix 8	C,D
P263L	9	15.4	A	loop	-
S318L	32	9.3	A	loop	-
P379L	19	18.1	B	loop	D
G420V	15	37.6	B	loop	E
G451C	30	12.9	C	loop	B,D
V457E	33	14.0	C	α-helix 17	A
T745R	18	25.5	D	β-sheet 33	-
R950P	16	41.2	E	β-sheet 47	-

· ***Relative activity vs wild-type.[S8] **There are five chains (A-E) in hLAMAN. (-) No interchain contact.

Table S2 : Puckering analysis of α-D-mannopyranose and derivatives from *D. melanogaster* GH38 α-mannosidase (UniProt id. Q24451) deposited in the PDB.[S2–S4]

PDB id.	Enzyme variant	Substrate	ϕ	θ	Q	Conformation
3BUQ	D204A	α-D-mannopyranose	305.50	5.21	0.57	$^4C_1$
3BUP	D341N	α-D-mannopyranose	308.78	54.01	0.56	E_5
3BVT	D204A	α-D-mannopyranose derivative	310.88	15.55	0.56	$^4C_1$
3BVU	D204A	α-D-mannopyranose derivative	310.79	20.25	0.58	$^4C_1$
3BVV	D204A	α-D-mannopyranose derivative	335.55	11.83	0.55	$^4C_1$
3BVW	D204A	α-D-mannopyranose derivative	311.33	16.62	0.56	$^4C_1$
3BVX	D204A	α-D-mannopyranose derivative	312.72	20.13	0.55	$^4C_1$
3CV5	D204A	α-D-mannopyranose derivative	337.56	13.70	0.57	$^4C_1$
3CZN	D204A	α-D-mannopyranose derivative	334.46	10.09	0.53	$^4C_1$
3CZS	D204A	α-D-mannopyranose derivative	313.42	27.55	0.47	$^4C_1$
5M7I*	D220N	α-D-mannopyranose-(1-6)-β-D-mannopyranose	331.28	79.72	0.67	$^oS_2$

* *C. perfringens* GH125 α-mannosidase (UniProt id. Q8XNB2).

Table S3 : Cremer-Pople angles ϕ and θ along the reaction pathway for wild-type and D74E variants.

Enzyme complex	ϕ (degree)	θ (degree)	Amplitude	Predominant conformation
wild-type ES	314.19 ± 12.20	70.33 ± 70.32	0.58 ± 0.05	E_5
wild-type ETS	293.66 ± 5.41	77.15 ± 3.18	0.59 ± 0.04	E_5 / $B_{2,5}$
wild-type GEI	274.98 ± 7.51	83.36 ± 2.96	0.73 ± 0.04	$^1S_5$
D74E ES	244.90 ± 27.26	88.50 ± 8.09	43.83 ± 3.83	$^1S_3$ / $^{1,4}B$
D74E ETS	279.73 ± 4.89	75.34 ± 3.76	41.28 ± 2.34	E_5 / $B_{2,5}$
D74E GEI	277.34 ± 4.52	83.42 ± 2.45	44.03 ± 2.17	$^1S_5$

Table S4 : Calculated SASAsub, SASApkt (Å^2) and substrate-positioning index (SPI) values along the QM/MM metadynamics trajectories.

Descriptor	wild-type	D74E	G153V	D159N	R229W	T745R
SASAsub	81.6 ± 10.7	91.2 ± 6.1	104.6 ± 8.1	107.2 ± 8.6	113.2 ± 8.4	114.8 ± 9.3
SASApkt	147.0 ± 15.6	111.8 ± 27.4	51.8 ± 17.2	98.4 ± 27.1	54.5 ± 15.4	37.54 ± 14.4
SPI	0.6	0.8	2.0	1.1	2.1	3.1

Table S5 : Distance difference (Δd, Å) between α-carbon atoms of residues H72, D74, D196, D319, H445, H446, and D447 in the equilibrated structures of the G153V, D159N, R229W, and T745R hLAMAN enzyme variants and equilibrated structure of wild-type hLAMAN after structural superimposition.

Enzyme	H72	D74	D196	D319	H445	H446	D447
wild-type	0.00	0.00	0.00	0.00	0.00	0.00	0.00
G153V	1.16	0.78	0.67	0.54	0.48	0.52	0.89
D159N	1.27	0.6	0.57	0.81	0.17	0.52	0.79
R229W	0.93	0.72	0.47	0.19	0.49	0.45	0.91
T745R	0.88	0.82	0.67	0.33	0.24	0.97	1.11

References

[S1] Stensland, H. M. F. R.; Frantzen, G.; Kuokkanen, E.; Buvang, E. K.; Klenow, H. B.; Heikinheimo, P.; Malm, D.; Øivind Nilssen amamutdb.no: A Relational Database for MAN2B1 Allelic Variants that Compiles Genotypes, Clinical Phenotypes, and Biochemical and Structural Data of Mutant MAN2B1 in -Mannosidosis. *Human Mutation* **2015**, *36*, 581–586.

[S2] Zhong, W.; Kuntz, D. A.; Ember, B.; Singh, H.; Moremen, K. W.; Rose, D. R.; Boons, G.-J. Probing the Substrate Specificity of Golgi -Mannosidase II by Use of Synthetic Oligosaccharides and a Catalytic Nucleophile Mutant. *Journal of the American Chemical Society* **2008**, *130*, 8975–8983.

[S3] Shah, N.; Kuntz, D. A.; Rose, D. R. Golgi -mannosidase II cleaves two sugars sequentially in the same catalytic site. *Proceedings of the National Academy of Sciences* **2008**, *105*, 9570–9575.

[S4] Alonso-Gil, S.; Males, A.; Fernandes, P. Z.; Williams, S. J.; Davies, G. J.; Rovira, C. Computational Design of Experiment Unveils the Conformational Reaction Coordinate of GH125 -Mannosidases. *Journal of the American Chemical Society* **2017**, *139*, 1085–1088.

[S5] Gaus, M.; Cui, Q.; Elstner, M. DFTB3: Extension of the Self-Consistent-Charge Density-Functional Tight-Binding Method (SCC-DFTB). *Journal of Chemical Theory and Computation* **2011**, *7*, 931–948.

[S6] Case, D. A.; Aktulga, H. M.; Belfon, K.; Ben-Shalom, I.; Brozell, S. R.; Cerutti, D. S.; Cheatham III, T. E.; Cruzeiro, V. W. D.; Darden, T. A.; Duke, R. E.; others *Amber 2021*; University of California, San Francisco, 2021.

[S7] Spela, M.; Bruno, D. G.; Santiago, A.-G.; Gibu, G.; Ulrika, F.; A, H. S.; Bojan, Z.;

Christoph, N.; A, S.-M. P. A new view of missense mutations in alpha-mannosidosis using molecular dynamics conformational ensembles. *ChemRxiv* **2024**,

[S8] Riise Stensland, H. M. F.; Klenow, H. B.; Nguyen, L. V.; Hansen, G. M.; Malm, D.; Nilssen, Identification of 83 novel alpha-mannosidosis-associated sequence variants: Functional analysis of MAN2B1 missense mutations. *Human Mutation* **2012**, *33*, 511–520.
